# Prediction of Unwanted Crystallization of Freeze-Dried Protein Formulations Using α-Relaxation Measurements

**DOI:** 10.3390/pharmaceutics15020703

**Published:** 2023-02-20

**Authors:** Sebastian Groël, Tim Menzen, Gerhard Winter

**Affiliations:** 1 Department of Pharmacy, Ludwig-Maximilians University Munich, 81377 Munich, Germany; 2 Coriolis Pharma Research GmbH, 82152 Martinsried, Germany

**Keywords:** amorphous pharmaceuticals, crystallization, differential scanning calorimetry (DSC), freeze-drying, isothermal microcalorimetry (IMC), molecular mobility

## Abstract

There is a lack of methods to predict the isothermal crystallization behavior of amorphous freeze-dried formulations stored below the glass transition temperature. This study applies isothermal microcalorimetry to predict long-term crystallization during product storage time. The relaxation curve of a fresh sample recorded within 12 h after lyophilization is correlated with the long-term crystallization time at the same temperature. Storage conditions of 25 °C and 40 °C are examined and five model formulations containing either sucrose or trehalose with different concentrations of an IgG_1_ antibody are investigated. The amorphous formulations were created by different freeze-drying processes only differing in their freezing step (random nucleation; additional annealing step of 1.5 h and 3 h, controlled nucleation; quench cooling). Samples that crystallized during the study time of 12 months showed a promising correlation between their relaxation time and crystallization behavior upon storage. Furthermore, the study shows that polysorbate 20 strongly accelerates crystallization of sucrose and that the freezing step itself has a strong impact on the relaxation phenomena that is not levelled out by primary and secondary drying.

## 1. Introduction

The amount of biological therapeutics has strongly increased in recent years [1]. Those entities face degradation problems during shipment and long-term storage when kept in their liquid formulation [2]. Thus, freeze- or spray-drying can be used to achieve long-term stability of these products upon removal of water [3,4]. To successfully freeze-dry those materials, sugars, such as sucrose or trehalose, are used as bulking agents and lyoprotectants [5]. Those excipients are able to protect and sustain the structure of biotherapeutics, including antibodies [6]. In particular, sugars can mimic water with their hydroxyl groups, thereby supporting the native conformation of the antibody upon lyophilization based on the water replacement theory [7,8]. To fulfil this stabilizing effect, the sugar has to stay amorphous during the storage time of the product [9,10]. However, during storage, crystallization of sugars can occur, even if the storage temperature is significantly below the glass transition temperature (T_g_) [11]. Therefore, it is important to avoid crystallization of the excipients during the life time of the pharmaceutical formulation [12]. Consequently, methods that predict crystallization in the early stage of development are needed to discover formulations and process conditions that are prone to result in crystallization upon storage, which frequently lasts up to 2 years or more [11,13,14].

Differential scanning calorimetry (DSC) is one method to estimate the crystallization tendency of a formulation [15]. With this method, the T_g_ and its onset as well as the energy of crystallization above T_g_ can be determined [16]. Of course, the onset of crystallization measured above T_g_ can be used as a surrogate for isothermal crystallization tendencies, but dominant reactions at high temperatures, especially above T_g_, are not always the leading reactions at lower temperatures below T_g_ [17]. Particularly in glasses where Arrhenius kinetics cannot be applied, this could lead to incorrect predictions [18].

Another method is X-ray Powder Diffraction (XRD), where an X-ray diffractogram of the freeze-dried product is recorded. An absence of clear reflection peaks is proof for a fully amorphous sample, as an ordered structure, such as a crystal, is necessary to diffract the beam in a well-defined angle [19]. This method is the standard approach to monitor the recent status of a sample, but has no predictive potential [20,21,22,23].

Below T_g_, structural relaxation phenomena are successfully used as general stability predicting factors for amorphous formulations [24,25]. One possibility to quantify relaxation processes is isothermal microcalorimetry (IMC) [26]. Here, α-relaxations can be estimated from a measured curve decay of a freshly prepared glass within a few days [27]. The curve shape can be fitted with equation 1 (see below) and expressed as the relaxation time $\tau^{\beta }$ [h]. The higher the energy excess is, the smaller the relaxation time $\tau^{\beta }$ [h] will be and the more degradation reactions, including crystallization, can happen [25]. Thus, the theory is to create amorphous formulations with as little energy excess as possible, which results in a high value of $\tau^{\beta }$. A detailed explanation and derivation of $\tau^{\beta }$ can be found elsewhere [25,28]. It should be pointed out that relaxation measurements are only suitable for process optimization with the same formulation. The excipients have a strong impact on product behavior, affecting multiple properties, and thus cannot be captured by α-relaxation measurements alone [28].

In earlier crystallization studies considering α-relaxations, single-component systems with either sucrose as the excipient [18,24] or non-protein active pharmaceutical ingredients (APIs), such as nifedipine [18,29], were utilized. Furthermore, most of the studies attempt to predict the onset of crystallization at the temperature of interest with relaxation measurements at other temperatures. In this study, in a first step, protein-free multi-component placebo formulations created by freeze-drying are investigated to verify that the prediction of crystallization in mixed formulations is possible at all. In a second step, an IgG_1_ antibody as API is added to the formulations. In both concepts, the systems were studied with IMC and after long-term storage.

In addition, a second question is targeted by this study. Most of the changes in the relaxation behavior of amorphous samples are explained with their different thermal history compared to a reference sample [30,31,32]. To investigate this, if the freezing step of a lyophilization process has an impact on the relaxation behavior of the final product, different freeze-drying protocols that only differ in their freezing step are applied (random nucleation; additional annealing step of 1.5 h and 3 h, controlled nucleation; quench cooling). Although the freezing step itself could be considered a thermal treatment, as the amorphous phase is shielded by the frozen water and no drying process is happening, it is possible that these effects are levelled off later by the thermal history introduced by primary and secondary drying.

Five different formulations are used to investigate if and how the prediction of isothermal crystallization is possible. Three formulations with a low antibody content of 2 mg/mL with different polysorbate 20 (PS20) concentrations ranging from 0–1.6 mg/mL, a formulation with a high antibody content of 50 mg/mL, and a formulation containing trehalose are studied. The different amounts of PS20 in the first three samples are considered to accelerate crystallization despite a low residual moisture content [33]. A high protein concentration of 50 mg/mL is used to investigate if the model is applicable for more challenging protein/sugar ratios and trehalose is utilized as another sugar with a higher T_g_ compared to sucrose.

## 2. Materials and Methods

### 2.1. Preparations of Formulations

An IgG1 antibody (LMU1) was utilized as a model protein. It was purified with a Sepharose HiTrap SP column (Cytiva, Marlborough, MA, USA) with an ÄKTA protein purification system (Cytiva, Marlborough, MA, USA) to remove polysorbate 20 (PS20) from the bulk starting material. The elution buffer was exchanged with a cross-flow filtration unit Minimate™ TFF capsule with an omega polyethersulfone (PES) membrane (MW 30,000 Da; Pall Corporation, New York, NY, USA) by using a 7-fold excess of 20 mM histidine buffer pH 5.5 (Alfa Aesar, Ward Hill, MA, USA). After the process, the concentration of LMU1 was determined with a NanoDrop™ 2000 UV photometer (Thermo Fisher Scientific, Waltham, MA, USA). Stock solutions of the further excipients, D-(+)-trehalose dihydrate (VWR International, Radnor, PA, USA), sucrose (Sigma-Aldrich, Steinheim, Germany), and polysorbate 20 (PS20) (Croda, Edison, NJ, USA), were created and mixed with the LMU1 solution to produce the formulations according to Table 1. Finally, the formulations were filtered using a 0.22-µm PES Sartolab® RF vacuum filter unit (Sartorius AG, Goettingen, Germany) before filling 1 mL solution in 2 R vials (MGlas AG, Muennerstadt, Germany). The vials were semi-stoppered with West 13-mm Lyo Nova Pure RS 1356 4023/50 G stoppers (West Pharmaceutical Services, Inc., Exton, PA, USA) and placed on a freeze-drying tray with two rows of placebo-filled vials as the outer radiation shield.

### 2.2. Freeze-Drying Protocols

**Figure 1:**
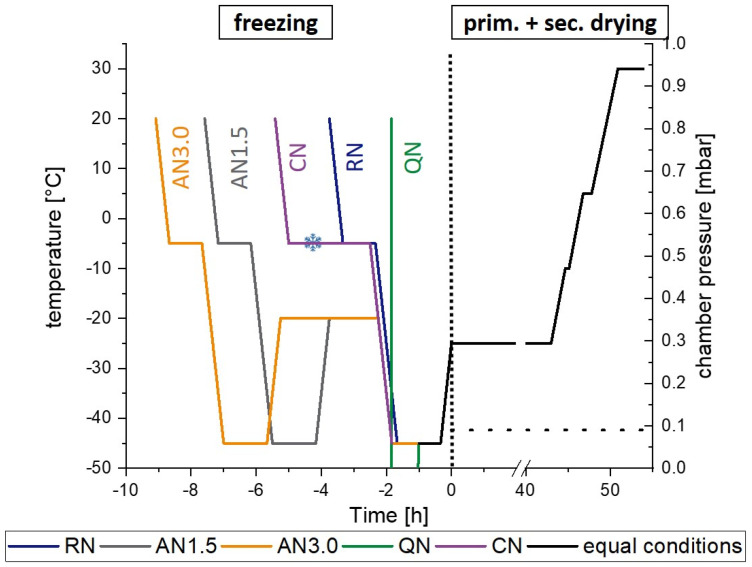
Graphical scheme of the different freezing processes. The snow flake in the CN curve indicates the introduction of ice crystals in the chamber after equilibration at −5 °C. The dotted line separates the freezing step from the drying process. RN, random nucleation; AN1.5, additional annealing step of 1.5 h; AN3.0, additional annealing step of 3.0 h; CN, controlled nucleation; QN, quench cooling. Solid lines represent the temperature in °C and the dashed line the chamber pressure.

Five freeze-drying processes only differing in their freezing steps are applied. The freezing protocols are presented in Table 2 and Figure 1.

To initiate primary drying, the temperature was ramped with 1 K/min from −45 °C to −25 °C and a pressure of 0.09 mbar was set. Primary drying was conducted for 48 h. For secondary drying, the shelves were ramped to 30 °C with a rate of 0.15 K/min including a holding time at −10 °C for 0.5 h and at 5 °C for 1 h. The final temperature of 30 °C was held for 4 h before the vials were stoppered under a nitrogen atmosphere. The freeze-drying was performed with a Christ ε2-6D laboratory-scale freeze-dryer (Martin Christ Gefriertrocknungsanlagen GmbH, Osterode am Harz, Germany) and a LyoCoN system (Martin Christ Gefriertrocknungsanlagen GmbH, Osterode am Harz, Germany) was utilized for a controlled nucleation process via ice fog. The complete freeze-drying protocols are displayed in Figure 1.

### 2.3. Residual Moisture Content

The residual moisture content in [% (*w/w*)] was determined by coulometric Karl Fischer Titration. The samples were ground with a spatula under a controlled atmosphere with a humidity <10% and 10–30 mg was transferred to a new 2 R Vial. The vials were heated in an oven to 100 °C, which allows the water to evaporate and be transferred by a dry gas stream into the measurement cell. The residual moisture content was calculated by dividing the measured mass of water by the transferred sample mass.

### 2.4. Differential Scanning Calorimetry

To determine the T_g_ [°C] and the $∆H_{r}\left(\infty \right)$ [J/g], a Mettler Toledo DSC 821e (Mettler Toledo, Gießen, Germany) differential scanning calorimeter was used. The values are obtained by using a modulated scanning mode with a heating rate of 2 K/min, a period of 2 min, and an amplitude of 1 K. To extract the data, the resulting signal was transformed in a reversing, non-reversing, and total curve. The reversing curve was used to define the values for the relaxation calculation whereas the total curve was used to investigate the crystallization peak. Sample preparation was conducted in a controlled atmosphere with a humidity <10%. In total, 5–15 mg sample were transferred to an aluminum crucible and hermetically sealed. An empty reference pan was prepared that stayed in the instrument for all measurements.

### 2.5. Isothermal Microcalorimetry (To Determine τ^β^)

A LKB 2277 Thermal Activity Monitor (TAM) equipped with 4 mL ampoule twin cylinders (2277-201) (TA Instruments, New Castle, DE, USA) was used to determine the α-relaxation times of the lyophilizates. The samples were transferred to stainless steel measuring ampoules in a controlled atmosphere with a humidity <10%. Approximately 150 mg pooled (from more than one glass vial to reach the required amount) samples were used. The reference ampoule remained empty. The measurement and reference ampoule were first lowered to the thermal equilibrium position and held for 15 min to allow equilibration. Both ampoules were then slowly lowered to the measurement position of the TAM. The experiments were performed at 25 °C and 40 °C. Data points were collected with an interval of 2 s for the first hour of the measurement and afterwards in a 10 s interval. The duration of the measurement was 12 h. The obtained curve was fitted in OriginPro 2019b with the modified stretch exponential function as suggested by Pikal and Kawakami (Equation (1)) [25]. The start parameters are set to $\tau_{0}=2$, $\tau_{1}=1$, and β=0.1 and further conditions are $\tau_{0}>\tau_{1}$ as well as 0<β≤1.

(1) $$P=277.8\times \frac{∆H_{r}\left(\infty \right)}{\tau_{0}}\times \left(1+\frac{\beta t}{\tau_{1}}\right)\times {\left(1+\frac{t}{\tau_{1}}\right)}^{\beta -2}\times \exp\left[-\left(\frac{t}{\tau_{0}}\right)\times {\left(1+\frac{t}{\tau_{1}}\right)}^{\beta -1}\right]\;\mathrm{with}\;∆H_{r}\left(\infty \right)=\left(T-T_{g}\right)\times {\;c}_{p}$$

### 2.6. Specific Surface Area

With a Brunauer-Emmet-Teller (BET) krypton gas adsorption instrument (Autosorb 1; 3P-Instruments, Odelzhausen, Germany), the specific surface area of the samples (SSA) was determined. The samples were ground with a spatula in a controlled atmosphere with a humidity <10% and 80–100 mg sample were transferred to measurement glass tubes. Outgassing was performed at room temperature for at least 2 h and the measured curve was fitted by the Autosorb 1 software.

### 2.7. X-ray Powder Diffraction

The absence of crystals in the freshly prepared samples was verified with a Rigaku MiniFlex benchtop XRD instrument (Rigaku Corporation, Tokyo, Japan). A copper anode at 40 kV and 15 mA was used to generate CuKα radiation (λ = 0.15417 nm). The freeze-dried samples were ground with a spatula under a controlled atmosphere with a humidity <10% and transferred to a silica sample holder. Powder diffraction was measured ranging from 3° to 60° 2θ in 0.02° measurement intervals with a speed of 10°/min.

### 2.8. Procedure to Correlate Crystallization with Relaxation Time τ^β^

To correlate the crystallization of the samples at 25 °C and 40 °C, the relaxation time τ^β^ [h] is plotted with the crystallization time during storage in [h]. To determine the latter, two procedures were carried out. For fast crystallizing samples (placebo formulations and 2 mg/mL_1.6PS_Suc at 40 °C), the samples were left in the IMC instrument until a crystallization peak occurred. The peak was confirmed as a crystallization event by subsequent DSC and XRD measurements. Here, the peak maximum was used as the crystallization time.

For all other samples that would block the IMC instrument too long, weekly DSC measurements of sample aliquots were performed. The changes in the crystallization peak above T_g_ (onset and enthalpy) were tracked and compared with the measurements performed the weeks before. The onset temperature of this peak was recorded and plotted. The reduction of the onset temperature above T_g_ also indicates an ongoing crystallization at the storage temperature of 25 °C below T_g_.

## 3. Results

### 3.1. Macroscopic and Microscopic Appearance

**Figure 2:**
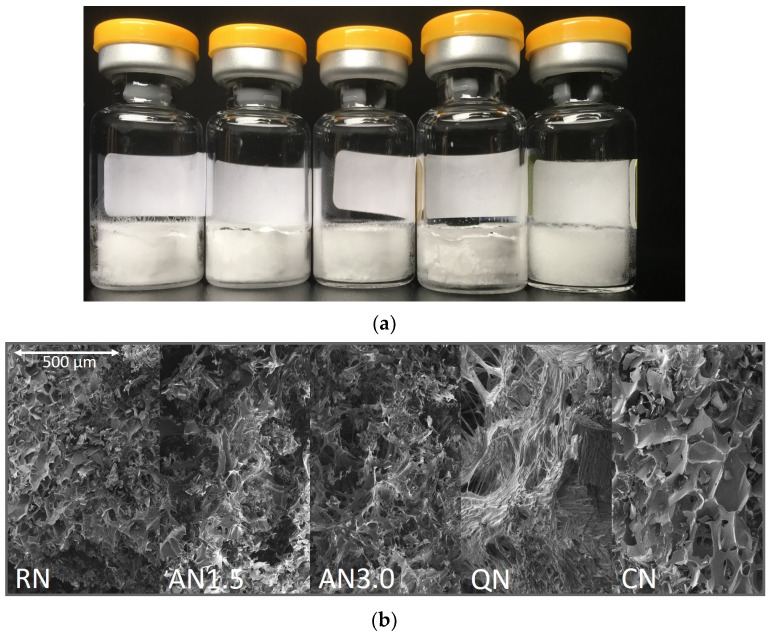
Representative optical appearance of 2 mg/mL_1.6PS_Suc samples: (**a**) macroscopic appearance and (**b**) scanning electron microscopy. Processes from left to right: RN, random nucleation; AN1.5, additional annealing step of 1.5 h; AN3.0, additional annealing step of 3.0 h; CN, controlled nucleation; QN, quench cooling.

Macroscopic and microscopic pictures of the 2 mg/mL_16PS_Suc are presented in Figure 2. They are representative for all formulations.

### 3.2. Residual Moisture

The residual moisture levels of the products are presented in Table 3. Additionally, the RN, QN, and CN processes from the 2 mg/mL_1.6PS_Suc formulation were equalized in their residual moisture content, as described by Lo Presti and Frieß [35]. The comparison with the untreated samples is presented in Table 4. Overall, the CN process leads to the highest residual moisture content whereas the QN process leads to products with the lowest level of residual moisture. The processes RN, AN1.5, and AN3.0 only slightly differ in their water content and no clear trend can be found. For the formulations containing sucrose and 2 mg/mL IgG_1_, no effect of the amount of PS20 on residual moisture can be observed. The 50 mg/mL_1.6PS_Suc and 2 mg/mL_0.4PS_Tre samples are drier after the RN, AN1.5, AN3.0, and QN processes compared to the other samples. Only after CN do they retain a water content comparable to the other formulations.

### 3.3. Specific Surface Area (SSA)

The values for the SSA of the samples can be found in Table 5. The results are similarly clustered as for residual moisture. The biggest differences between processes are obtained between CN and QN, with CN having the lowest and QN the highest SSA. The other processes result in quite similar surfaces. Between formulations, the biggest differences can be found comparing the 2 mg/mL_0.4PS_Tre formulation and the 50 mg/mL_1.6PS_Suc formulation with the remaining formulations, with all 2 mg/mL_Suc samples having lower SSA values for the RN, AN1.5, and AN3.0 process. Additionally, the SSA for 50 m/mL_1.6PS_Suc formulations were significantly higher in QN and lower in CN than all other formulations.

### 3.4. Differential Scanning Calorimetry Results

Table 6 shows the T_g_ values of the freeze-dried formulations. The complete DSC results with $∆c_{p}$, $∆H_{r}\left(\infty \right)$ at 25 °C and 40 °C as well as the onset and enthalpy of crystallization are not presented. They are considered in the calculation of the relaxation data and are presented in Appendix A to improve the clarity of the publication.

### 3.5. X-ray Powder Diffraction

Absence of crystallinity was shown by XRD for all freshly freeze-dried formulations. The onset of crystallization estimated by IMC and DSC was reviewed by XRD measurements.

### 3.6. Isothermal Microcalorimetry

**Figure 3:**
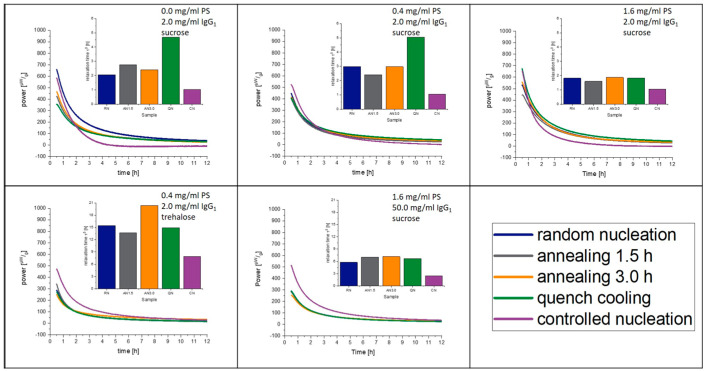
Relaxation curves obtained from IMC at 40 °C over 12 h.

**Figure 4:**
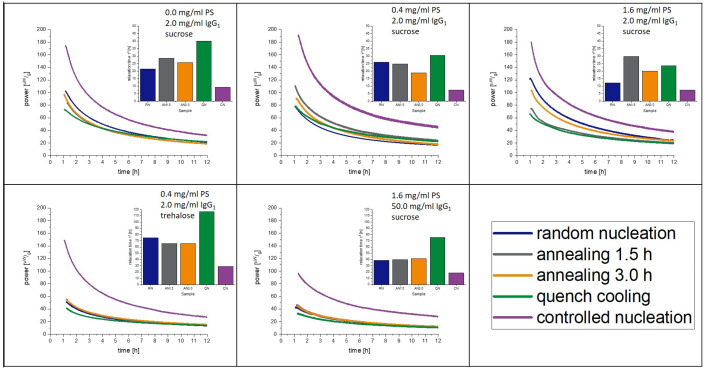
Relaxation curves obtained from IMC at 25 °C over 12 h.

The measured relaxation curves as well as the calculated τ^β^-values are shown in Figure 3 and Figure 4 for 40 °C and 25 °C, respectively. The CN-samples show the shortest relaxation time in all samples over all tested temperatures. The order of relaxation times of the remaining samples varies depending on temperature and formulation with the QN process having, in most cases, the longest relaxation time.

## 4. Discussion

All freeze-drying processes with the different freezing steps resulted in acceptable dry products. Results from product temperature monitoring can be found in Appendix B. The microscopic and macroscopic appearance, as well as the SSA, indicated that the freezing steps resulted in products with different morphology, as desired. It was expected for the controlled nucleation (CN) samples to have the largest pores (Figure 2), but lower overall surface area [36,37]. During drying, bigger pores remain, which lead to faster primary drying, but to less effective secondary drying as the SSA is relatively low [38,39]. In the QN process, on the other hand, plenty of very small ice nuclei arise [40]. Thus, the QN process leads to very small pores and an overall high SSA (Table 5). The RN process can be considered as in-between and ends up with an intermediate SSA. The AN1.5 and AN3.0 processes increase the time for ice nuclei to grow but this trend was not observed in our samples [41,42]. The reason might be that the annealing conditions of −20 °C with a time of only 1.5 or 3.0 h were too short and not high enough in temperature to allow stronger effects. Recent trends in the community are suggesting more “aggressive” annealing conditions close to the eutectic temperature at −4 °C over more than 5 h [43], but were not yet applied in our study.

The trends seen for the structure of the cakes are clearly connected with the residual moisture content of the samples. After primary drying, about 10–15% bound water remains in the amorphous phase of the formulation, which is then removed in secondary drying [44,45]. Here, formulations with a higher SSA will dry much faster. Therefore, applying the same secondary drying conditions leads to a higher residual moisture level in the CN samples compared to the QN samples. The differences in residual moisture impact α-relaxations [31] as well as crystallization [46]. When searching for a correlation of α-relaxation with crystallization, differences in residual moisture should not matter as both effects should be equally influenced if a correlation exists. However, to answer the question what impact the freezing step has on α-relaxations is a challenge. Water, as a plasticizer, strongly affects the T_g_ [31] of the freeze-dried cake and, with this, also α–relaxations. For this reason, it is not possible, without further intervention, to separate the effects of the freezing step and residual moisture. Thus, the changes in measured α-relaxations could emerge from the changes in residual moisture alone. To overcome this challenge, two approaches can be applied. The secondary drying for samples with a lower SSA could be prolonged or an equalizing procedure by moisture adaption (as described by Lo Presti & Frieß) could be applied [35]. The first option would change the thermal history and can therefore not be utilized. The second option is too time-consuming to be applied for an entire 12-month stability study sample set. Therefore, a pre-test study was performed on the 1.6PS_Suc placebo formulation without protein, considered as a sample with a high and fast crystallization tendency. This approach shall give insight whether the correlation of α-relaxation with crystallization is possible.

### 4.1. Placebo Formulation Pre-Test

**Figure 5:**
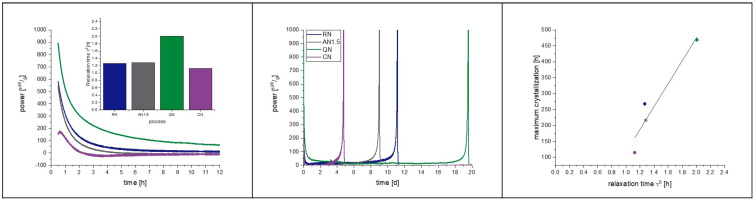
Placebo pre-tests at 40 °C to examine the correlation of crystallization with α-relaxation and the effect of the freezing step on the latter. The left picture presents a zoomed in area of the IMC measurement to focus on the relaxation curves, which are converted to a τ^β^-value. The middle graph shows the full view of the measurement including the crystallization of the samples. On the right, the correlation of relaxation time with crystallization maximum of the IMC curves is presented. The correlation coefficient of the fit is r^2^ = 0.88. Random nucleation (blue), additional annealing step of 1.5 h (grey), quench cooling (green), controlled nucleation (purple).

Figure 5 shows the result of this pre-test. A similar approach had already been published but had not been evaluated concerning the crystallization tendency [28]. The residual moisture of the samples is around 1.3 ± 0.2% after moisture equalization and is considered as equal [28,31]. It can be seen in Figure 5 that the freezing step has a clear influence on the relaxation, with CN having the shortest relaxation time and QN the longest. RN and AN1.5 are in between. The annealing time of 1.5 h at −20 °C was not long enough to introduce stronger effects. Thus, in the following main study, an annealing step of 3.0 h was added. Nevertheless, the crystallization of the samples occurs qualitatively exactly in the order of the relaxation times, with CN being the first sample to crystallize, followed by AN1.5, RN, and with QN being the last. A reasonable linear correlation between relaxation times and onset of crystallization with r^2^ = 0.88 can be found. Moreover, it is remarkable that the relaxation curve of CN samples shows a different shape than the other curves. In the beginning, besides the expected crystallization peak, a second exothermal peak maximum is obtained at 0.58 h followed by an endothermal valley lasting from 2–5 days of measurement. A similar observation was made for foam-dried [30] samples as well as for tempered and CN samples [28]. The use of controlled ice nucleation seems to introduce similar effects in the lyophilized matrix as a tempering procedure or the heat applied during foam drying.

Overall, the freezing step itself and residual moisture are two separate factors influencing the relaxation behavior and the impact of the freezing step is not levelled off by the drying step. Importantly, a correlation of α-relaxation with crystallization was observed and shall now be investigated more deeply in protein-containing samples.

### 4.2. Protein-Containing Samples Equalized for Residual Moisture

Based on the data in Section 4.1, the residual moisture was only adapted for a small group of samples to see if a correlation between relaxation and crystallization also exists in protein-containing matrices. The moisture adapted samples are 2.0 mg/mL_1.6PS_Suc processed by freeze-drying with RN, CN, and QN. Table 4 shows the residual moisture data of these samples before and after adaption.

**Figure 6:**
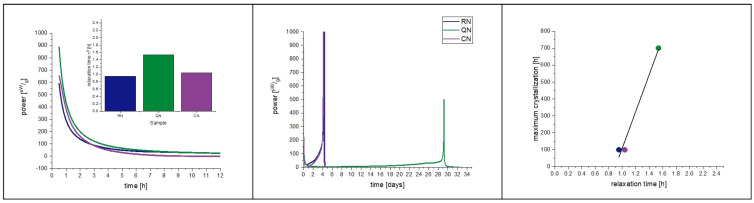
Moisture equalized samples from the 2 mg/mL_1.6PS_Suc formulation at 40 °C, correlation of crystallization with α-relaxation, and the effect of the freezing step. The left picture presents a zoomed in area of the IMC measurement to focus on the relaxation curves, which are converted to a τ^β^-value. The middle graph shows the full view of the measurement including the crystallization of the samples. On the right, the correlation of relaxation time with crystallization maximum of the IMC curves is presented. The correlation coefficient of the fit is r^2^ = 0.96. Random nucleation (blue), quench cooling (green), controlled nucleation (purple).

**Figure 7:**
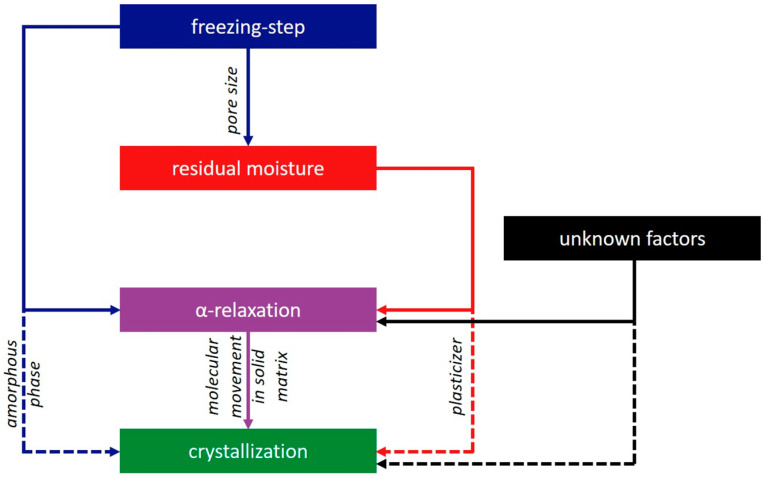
Graphical scheme of the correlation of the investigated parameters. Solid lines were verified by the pre-tests; dashed lines are effects that seem to be proportional to the connected solid line according to the pre-tests.

With protein formulations, a correlation coefficient of r^2^ = 0.92 can be obtained by a linear fit of relaxation with crystallization time. With moisture adaption, the correlation coefficient increased to a value of r^2^ = 0.96 (Figure 6). We are aware of the fact that a linear correlation with three data points is statistically not robust. However, the pre-tests with placebo and protein-containing samples with moisture adaption were performed to generate a first insight into the relation of relaxation, crystallization, freezing step, and residual moisture (Figure 7). For this purpose, a limited number of samples was sufficient and justified to set up a bigger study with more processes and formulations. Before we delve into the findings of the main study, we want to summarize what has been found so far:

- The freezing step influences the residual moisture directly by determining the pore size of the formulation [36,37]. Furthermore, the freezing step influences α-relaxation directly by changes in the amorphous phase as well as indirectly by the residual moisture. Both effects are independent from each other.
- The residual moisture influences the α-relaxation and crystallization directly by plastization of the solid phase [31]. The effect on α-relaxation and crystallization is proportional.
- α-relaxation and crystallization below T_g_ correlate not only in single-component but also in multi-component systems. The addition of 2 mg/mL of protein changes the crystallization behavior of the product (Figure 5 and Figure 6), but the correlation of α-relaxation and crystallization remains.

All in all, several effects that influence the crystallization tendency of a dry amorphous cake below T_g_ also influence the α-relaxation in a proportional way. Thus, to determine the crystallization tendency of a formulation, it appears suitable to use α-relaxation as the leading parameter. The hypothesis is that α-relaxation is the only factor that is changed by all factors that affect crystallization and can display those changes introduced by a freeze-drying process in a predictive measurement.

### 4.3. Protein-Containing Samples

Although the correlation of α-relaxations with crystallinity is the focus of the study, three further functional aspects can be observed from the investigations of the protein samples. One is the effect of PS20 on the crystallization of sucrose in lyophilized solids, which can be analyzed by the 2gl_Suc formulations with the different amounts of PS20. Next, the effect of protein concentration on α-relaxations can be attained by comparing 2gl_1.6PS_Suc with 50gl_1.6PS_Suc. Last, the influence of the sugar itself on τ^β^ and the T_g_ by comparing the 2gl_0.4_Suc with 2gl_0.4PS_Tre can be obtained.

During the investigation period of 12 months, the 2 mg/mL_1.6PS_Suc samples crystallized isothermally at 25 °C and 40 °C, respectively. The 2 mg/mL_0.4PS_Suc formulation from the CN process crystallized at 40 °C, but not at 25 °C. All other process/formulation combinations led to samples that stayed amorphous (Table 7).

From these observations we conclude the following. First, PS20 accelerates crystallization of sucrose. All samples with 2 mg/mL protein and 1.6 mg/mL PS20 crystallized. The only sample with a PS20 concentration below 1.6 mg/mL that crystallized was the 2 mg/mL_0.4PS_Suc formulation from the CN process at 40 °C storage. Here, the significantly higher residual moisture resulting from the CN process was the reason (Table 3). In contrast, the corresponding sample without PS20 was still amorphous. The mechanism behind the acceleration of crystallization itself is not clear. Factors such as T_g_, SSA, and residual moisture were ruled out as they are approximately the same within all the respective samples. The PS20 concentrations of 0.4 mg/mL and 1.6 mg/mL are both far above the critical micelle concentration (CMC) of PS20 [47,48,49]. It is possible that micelles of PS20 could act as crystallization nuclei for sucrose. More micelles present in the 1.6 mg/mL PS20 formulation could consequently lead to faster crystallization.

Second, even small changes in excipient composition can change the physical properties of the matrix and some are not detectable with α-relaxation measurements. Concerning the relaxation time τ^β^, the 2 mg/mL_0.0PS_Suc sample and the 2 mg/mL_1.6_PS_Suc sample are very similar (relaxation time of 1.03 h and 1.04 h, respectively), but evidently, they crystallize at different timepoints upon storage.

Third, a high protein concentration prevents crystallization of the matrix. Whether proteins act as a “binding partner” to PS20 and inhibit the crystallization accelerating effect of the surfactant or whether it is a result of the higher T_g_ (Table 6, approx. + 20 °C for 50 mg/mL compared to 2 mg/mL) cannot be answered.

**Figure 8:**
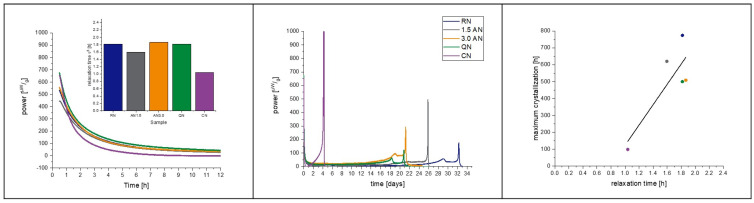
Samples from the 2 mgmL_1.6PS_Suc formulation at 40 °C; correlation of crystallization with α-relaxation, and the effect of the freezing step. The left picture presents a zoomed in area of the IMC measurement to focus on the relaxation curves, which are converted to a τ^β^-value. The middle graph shows the full view of the measurement including the crystallization of the samples. On the right, the correlation of relaxation time with crystallization maximum of the IMC curves is presented. The correlation coefficient of the fit is r^2^ = 0.58. Random nucleation (blue), additional annealing step of 1.5 h (grey), additional annealing step of 3.0 h (orange), quench cooling (green), controlled nucleation (purple).

Figure 8 shows the correlation of α-relaxation and crystallization of the 2 mg/mL_1.6PS_Suc samples at 40 °C. In all formulations, except CN, the rise of the crystallization peak started very slowly, and two peaks are observed. The double peaks (maxima 29.0 d and 32.3 d for RN; 21.8 d and 25.9 d for AN1.5; 19.0 d and 21.2 d for AN3.0; 18.3 d and 20.8 d for QN) arise through the pooling of samples from different vials to reach the minimum amount of 150 mg for IMC measurement. There are certain differences of 2 to 5 days between single vials regarding crystallization time. It is assumed that such differences between samples always exist, but in this case, were carved out through the slow crystallization. The crystallization in Section 4.2 was fast and differences within the pooled samples were so small that they were not observed. In long-term stability studies with drawing times of 1 and 3 months, a crystallization time difference of 5 days would of course not be detectable.

As the onset of crystallization is not directly detectable in the IMC, the maximum crystallization is used to correlate relaxation with crystallization. With r^2^ = 0.58, the quality of the fit is not as good as in the pre-test, but still indicates a strong correlation. The relaxation times τ^β^ are very close to each other, ranging from approximately 1–5 h. Within the sample set of 2 mg/mL_1.6PS_Suc, this means a 1 h difference in relaxation time τ^β^ corresponds to a shift of 601.35 h (25 days) in crystallization.

Nevertheless, at least a qualitative correlation was found, which is very useful if samples from different processes shall be compared for maintaining their amorphous state during long-term storage.

**Figure 9:**
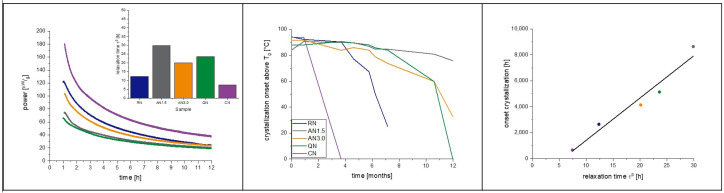
Samples from the 2 mgmL_1.6PS_Suc formulation at 25 °C; correlation of crystallization with α-relaxation, and the effect of the freezing step. The left picture presents a zoomed in area of the IMC measurement to focus on the relaxation curves, which are converted to a τ^β^-value. The middle graph shows the crystallization onset of the samples at 25 °C determined with DSC measurement using the onset of crystallization above T_g_. On the right, the correlation of relaxation time with crystallization onset is presented. The correlation coefficient of the fit is r^2^ = 0.94. Random nucleation (blue), additional annealing step of 1.5 h (grey), additional annealing step of 3.0 h (orange), quench cooling (green), controlled nucleation (purple).

Taking the same samples at a 25 °C storage temperature into account, the order of sample crystallization changed (Figure 9).

Comparing the prediction performance of the IMC with the relaxation time τ^β^ method at 25 °C with 40 °C qualitatively, it must be noted that at 40 °C, only one sample was correctly predicted. In contrast, at 25 °C, all five samples were correctly predicted in the order of crystallization with a correlation coefficient of r^2^ = 0.94.

In summary, at 40 °C, relaxation times are so close to each other that despite good resolution of the instrument, discrimination between the very small differences in relaxation of the samples is at its limits. At 25 °C, the resolution is high enough to measure the differences.

### 4.4. Comparison of IMC and DSC as Methods for Crystallization Prediction

**Figure 10:**
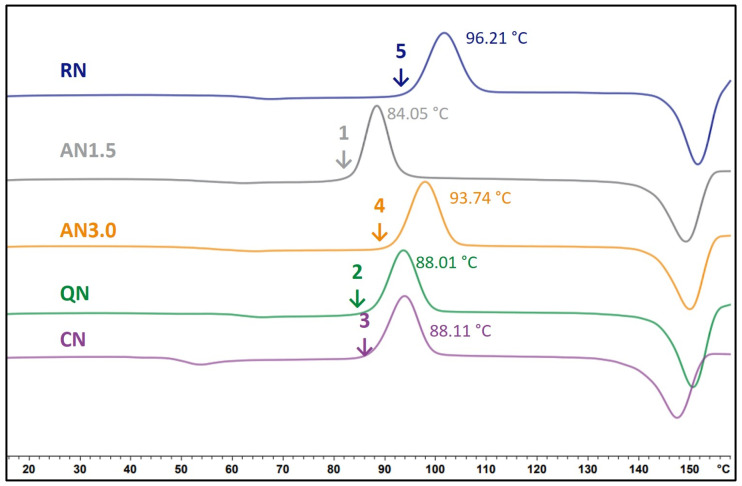
DSC thermograms of the 2 mg/mLg_1.6PS_Suc formulations. Processes as indicated at the corresponding curve. The temperature in °C displays the onset of crystallization temperature. The numbers rank the order of crystallization onset, starting with the first sample that starts to crystallize.

Comparing the results of the IMC and the DSC, the following can be concluded. At 25 °C, the IMC data has a much better performance than the DSC-based methods. Through the theory of global α-relaxations, it is understood that the IMC method detects more factors that influence crystallization than DSC measurements do. Furthermore, the behavior of glasses significantly changes at T_g_ making correlations and predictions from experiments above T_g_ with crystallization below T_g_ difficult [11,17]. As presented, the order of crystallization of the different samples changed from the 40 °C storage to 25 °C storage temperature. Simultaneously, the order of relaxation times also changed within the corresponding samples. The relaxation method is hence able to detect these changes whereas DSC, in contrast, shows the same; one DSC curve must be used for predictions at 25 °C and 40 °C storage (Figure 10). Isothermal microcalorimetry and α-relaxation determination is therefore a useful tool to predict crystallization under different storage conditions. It must be kept in mind, though, that for the calculation of τ^β^-values, T_g_ values from DSC measurements are necessary.

### 4.5. Influence of PS20 on Relaxation and Crystallization

**Figure 11:**
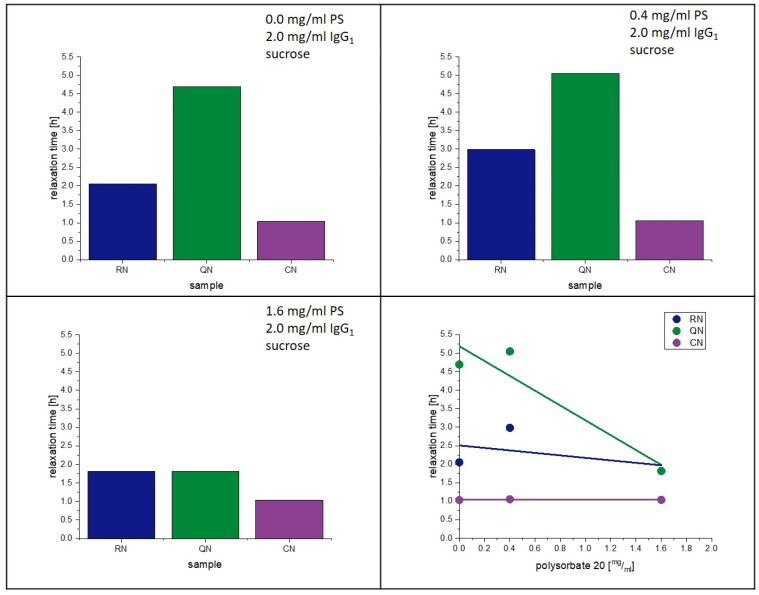
Influence of polysorbate on relaxation at 40 °C.

**Figure 12:**
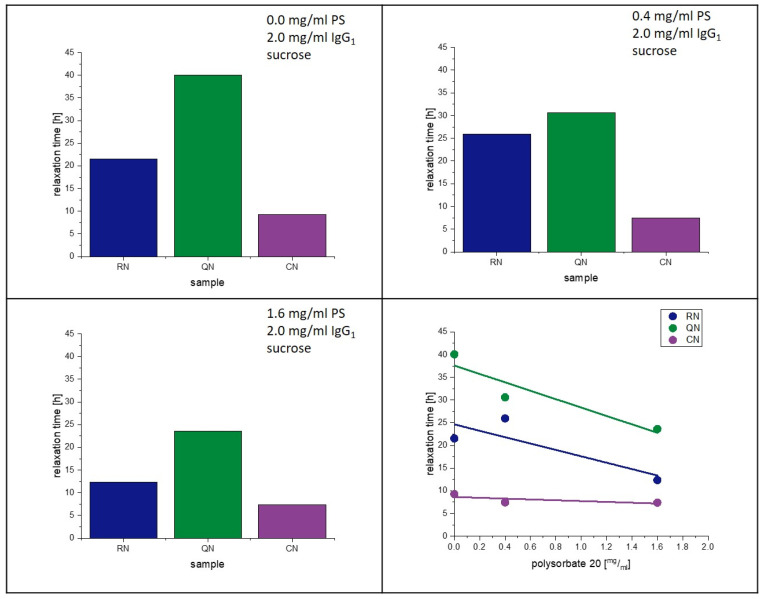
Influence of polysorbate on relaxation at 25 °C.

The present study supports the observation of Vollrath et al. [33]. That PS20 accelerates the crystallization of lyophilized amorphous sucrose matrices. Reasons for this phenomenon were already discussed above. Moreover, an influence of PS20 on the relaxation behavior had been observed in earlier studies [28]. Figure 11 and Figure 12 show the change in relaxation times depending on the PS20 concentration. An increase in the PS20 concentration led to a reduction in relaxation time τ^β^. This effect is more prone in the QN samples and least in the CN samples. From the aspect that PS20 is surface active, a connection with surface effects is very plausible, as QN samples have the highest and CN samples the lowest SSA. As seen in Table 6, the T_g_ temperatures of the formulation did not significantly change with change in PS20 concentration, but the relaxation time did. Thus, it can be concluded that PS20 has a matrix effect on the formulation that is not captured in the T_g_.

## 5. Conclusions

It is possible to predict the isothermal crystallization of protein-containing freeze-dried formulations below T_g_ by measuring the α-relaxation time, τ^β^, when certain conditions are fulfilled. Only different processes of the same formulation can be compared. A comparison of different formulations is not possible because the impact of formulation compositions overpowers the impact of the process on the matrix’s relaxation behavior. PS20 accelerates the crystallization of sucrose-based lyophilizates. The mechanism was not part of the study; however, it is important to emphasize that higher amounts of PS20 should be used with care. The presented method is not intended to replace DSC measurements and shall be used as an add-on. IMC could predict, at an early development stage, whether a certain lyophilization process would lead to a more or less stable product concerning crystallization over months. The standard analytics of DSC or XRD used during real-time stability studies are unable to deliver such predictive information.

## Figures and Tables

**Table 1:** Used formulations. Concentrations given in mg/mL.

Excipient	2 mg/mL 00PS_Suc	2 mg/mL 04PS_Suc	2 mg/mL 16PS_Suc	50 mg/mL 16PS_Suc	2 mg/mL 04PS_Tre
LMU1	2	2	2	50	2
sucrose	79.45	79.45	79.45	79.45	-
trehalose	-	-	-	-	79.45
PS20	-	0.4	1.6	1.6	0.4

**Table 2:** Applied freezing protocols during the lyophilization process.

Process	Step Number [#]	Shelf Temperature [°C]	Hold Time [h]	Ramp Rate of the Shelf towards the Next Step [K/min]
RN	1	20	-	−1
	2	−5	1.00	−1
	3	−45	1.50	*
AN1.5	1	20	-	−1
	2	−5	1.00	−1
	3	−45	1.50	+1
	4	−20	1.50	−1
	5	−45	1.50	*
AN3.0	1	20	-	−1
	2	−5	1.00	−1
	3	−45	1.50	+1
	4	−20	3.00	−1
	5	−45	1.50	*
CN	1	20	-	−1
	2	−5	1.00	†
	3	−5	2.00	−1
	4	−45	1.50	*
QN	1	20	-	quenching
	2	−196 ^‡^	0.03	-
	3	−45	1.500	*

* indicates the start of primary drying.† Indicates the controlled nucleation process by introduction of ice fog. ‡ estimated temperature of a liquid nitrogen bath [34].

**Table 3:** Residual moisture levels of the freeze-dried formulations in [% *w*/*w*]. *n* = 2, values represent the mean value and the corresponding standard deviation.

Excipient	2 mg/mL 00PS_Suc	2 mg/mL 04PS_Suc	2 mg/mL 16PS_Suc	50 mg/mL 16PS_Suc	2 mg/mL 04PS_Tre
RN	1.04 ± 0.03	0.84 ± 0.06	0.70 ± 0.06	0.31 ± 0.02	0.28 ± 0.05
AN1.5	0.90 ± 0.02	0.77 ± 0.07	0.85 ± 0.13	0.39 ± 0.04	0.36 ± 0.02
AN3.0	0.85 ± 0.00	0.93 ± 0.04	0.85 ± 0.01	0.35 ± 0.02	0.33 ± 0.03
QN	0.41 ± 0.04	0.49 ± 0.14	0.55 ± 0.02	0.17 ± 0.06	0.13 ± 0.01
CN	1.45 ± 0.10	1.42 ± 0.12	1.35 ± 0.02	1.34 ± 0.01	1.59 ± 0.22

**Table 4:** Comparison of residual moisture levels of the original and moisture equilibrated 2 mg/mL_1.6PS_Suc samples in [% *w*/*w*]. *n* = 2, values represent the mean value and the corresponding standard deviation.

Process	Original	Moisture Equilibrated
RN	0.70 ± 0.06	1.30 ± 0.14
QN	0.55 ± 0.02	1.15 ± 0.16
CN	1.35 ± 0.02	1.36 ± 0.12

**Table 5:** Specific surface area of the created formulations in [m^2^g^−1^]. *n* = 1, two samples were pooled in one measuring tube to reach the needed sample mass.

Excipient	2 mg/mL 00PS_Suc	2 mg/mL 04PS_Suc	2 mg/mL 16PS_Suc	50 mg/mL 16PS_Suc	2 mg/mL 04PS_Tre
RN	0.455	0.552	0.563	0.714	0.665
AN1.5	0.404	0.594	0.549	0.747	0.739
AN3.0	0.528	0.574	0.506	0.726	0.616
QN	1.080	0.982	0.914	2.270	0.982
CN	0.328	0.313	0.241	0.157	0.389

**Table 6:** Glass transition temperatures of the freeze-dried formulations [°C]. *n* = 2, values represent the mean value and the corresponding standard deviation.

Excipient	2 mg/mL 00PS_Suc	2 mg/mL 04PS_Suc	2 mg/mL 16PS_Suc	50 mg/mL 16PS_Suc	2 mg/mL 04PS_Tre
RN	60.21 ± 0.04	62.26 ± 2.73	62.27 ± 2.93	80.32 ± 0.04	104.46 ± 0.09
AN1.5	60.09 ± 0.03	64.10 ± 1.41	60.19 ± 1.74	84.14 ± 0.24	104.09 ± 0.04
AN3.0	60.16 ± 0.04	62.09 ± 2.74	60.01 ± 2.47	80.31 ± 0.42	104.08 ± 0.16
QN	72.03 ± 0.06	66.20 ± 3.19	64.05 ± 3.06	84.14 ± 0.42	106.51 ± 0.23
CN	48.98 ± 0.08	50.45 ± 2.67	48.29 ± 3.11	63.82 ± 0.70	100.11 ± 0.11

**Table 7:** Sample status after 12 months. X = crystallized during storage; - = still amorphous after 12 months.

Process	2 mg/mL 00PS_Suc		2 mg/mL 04PS_Suc		2 mg/mL 16PS_Suc		50 mg/mL 16PS_Suc		2 mg/mL 04PS_Tre	
	25 °C	40 °C	25 °C	40 °C	25 °C	40 °C	25 °C	40 °C	25 °C	40 °C
RN	-	-	-	-	X	X	-	-	-	-
AN1.5	-	-	-	-	X	X	-	-	-	-
AN3.0	-	-	-	-	X	X	-	-	-	-
QN	-	-	-	-	X	X	-	-	-	-
CN	-	-	-	X	X	X	-	-	-	-

**Figure A1:**
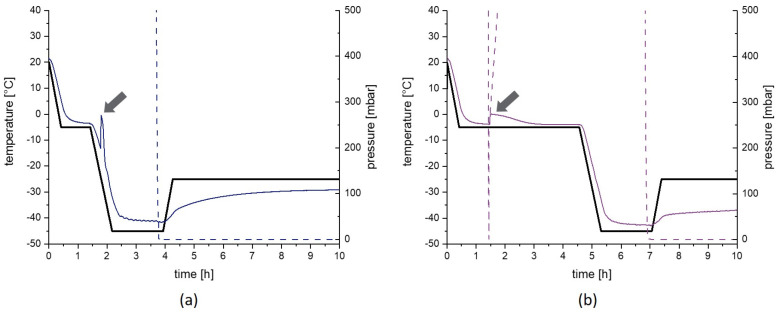
Monitoring of freezing of the products. The first 10 h of the random nucleation process (**a**) and the controlled nucleation process (**b**) are presented. The black line represents the temperature set point for the shelfs, the colored solid lines the actual product temperature and the dashed line the chamber pressure. Arrows demonstrate the ice nucleation process. The pressure drop and rise at approximately 1.5 h in the controlled nucleation is needed to introduce the ice fog in the chamber.

## Data Availability

Data are contained within the article.

## Appendix A

The following Table A1, Table A2, Table A3, Table A4 and Table A5 display the further calorimetric results that were left out in the main section of the publication due to clarity.

**Table A1:** Calorimetric data of the 2gl_0.0PS_Suc [°C]. *n* = 2, values represent the mean value and the corresponding standard deviation.

	RN	AN1.5	AN3.0	QN	CN
T_g_ [°C]	60.21 ± 0.04	60.09 ± 0.03	60.16 ± 0.04	72.03 ± 0.06	48.98 ± 0.08
Δc_p_ [Jg^−1^K^−1^]	0.55 ± 0.02	0.42 ± 0.05	0.52 ± 0.08	0.46 ± 0.04	0.51 ± 0.06
$∆H_{\infty }\left(25\;{}^{\circ}C\right)$ [Jg^−1^]	19.21 ± 0.72	14.81 ± 1.76	18.18 ± 2.82	21.45 ± 1.90	12.33 ± 1.46
$∆H_{\infty }\left(40\;{}^{\circ}C\right)$ [Jg^−1^]	11.02 ± 0.41	8.48 ± 1.01	10.42 ± 1.62	14.61 ± 1.29	4.62 ± 0.55
crystallization onset [°C]	97.20 ± 2.57	100.10 ± 0.42	101.65 ± 0.25	119.90 ± 0.28	91.96 ± 1.10
crystallization energy [Jg^−1^]	70.48 ± 0.95	66.47 ± 0.30	71.20 ± 7.71	62.66 ± 2.31	64.15 ± 1.09

**Table A2:** Calorimetric data of the 2gl_0.4PS_Suc [°C]. *n* = 2, values represent the mean value and the corresponding standard deviation.

	RN	AN1.5	AN3.0	QN	CN
T_g_ [°C]	62.26 ± 2.73	64.10 ± 1.41	62.09 ± 2.74	66.20 ± 3.19	50.45 ± 2.67
Δc_p_ [Jg^−1^K^−1^]	0.52 ± 0.12	0.62 ± 0.02	0.560 ± 0.03	0.48 ± 0.07	0.52 ± 0.10
$∆H_{\infty }\left(25\;{}^{\circ}C\right)$ [Jg^−1^]	19.23 ± 5.21	24.04 ± 0.95	20.73 ± 2.07	19.92 ± 3.79	13.14 ± 3.31
$∆H_{\infty }\left(40\;{}^{\circ}C\right)$ [Jg^−1^]	11.49 ± 3.12	14.82 ± 0.58	12.35 ± 1.23	12.67 ± 2.41	5.40 ± 1.36
crystallization onset [°C]	103.87 ± 5.53	106.15 ± 2.84	96.80 ± 1.31	99.23 ± 6.61	87.15 ± 1.10
crystallization energy [Jg^−1^]	66.75 ± 1.54	74.32 ± 0.29	69.41 ± 1.00	68.74 ± 1.10	62.69 ± 1.10

**Table A3:** Calorimetric data of the 2gl_1.6PS_Suc [°C]. *n* = 2, values represent the mean value and the corresponding standard deviation.

	RN	AN1.5	AN3.0	QN	CN
T_g_ [°C]	62.27 ± 2.93	60.19 ± 1.74	60.01 ± 2.47	64.05 ± 3.06	48.29 ± 3.11
Δc_p_ [Jg^−1^K^−1^]	0.54 ± 0.02	0.47 ± 0.03	0.57 ± 0.01	0.29 ± 0.01	0.44 ± 0.01
$∆H_{\infty }\left(25\;{}^{\circ}C\right)$ [Jg^−1^]	20.20 ± 1.85	16.64 ± 1.54	20.04 ± 0.90	11.13 ± 0.92	10.14 ± 0.93
$∆H_{\infty }\left(40\;{}^{\circ}C\right)$ [Jg^−1^]	12.07 ± 1.10	9.55 ± 0.88	11.45 ± 0.51	6.85 ± 0.57	3.61 ± 0.33
crystallization onset [°C]	96.21 ± 2.83	84.05 ± 1.16	93.74 ± 0.26	88.01 ± 1.54	88.11 ± 2.86
crystallization energy [Jg^−1^]	64.79 ± 0.04	57.62 ± 1.23	62.42 ± 1.23	60.85 ± 0.98	63.68 ± 3.02

**Table A4:** Calorimetric data of the 50gl_1.6PS_Suc [°C]. *n* = 2, values represent the mean value and the corresponding standard deviation.

	RN	AN1.5	AN3.0	QN	CN
T_g_ [°C]	80.32 ± 0.04	84.14 ± 0.24	80.31 ± 0.42	84.14 ± 0.42	63.82 ± 0.70
Δc_p_ [Jg^−1^K^−1^]	0.30 ± 0.01	0.34 ± 0.10	0.31 ± 0.02	0.33 ± 0.02	0.40 ± 0.02
$∆H_{\infty }\left(25\;{}^{\circ}C\right)$ [Jg^−1^]	16.71 ± 0.63	20.05 ± 5.97	16.76 ± 1.10	19.58 ± 1.28	15.37 ± 0.72
$∆H_{\infty }\left(40\;{}^{\circ}C\right)$ [Jg^−1^]	12.18 ± 0.46	14.96 ± 4.46	12.21 ± 0.80	14.61 ± 0.96	9.43 ± 0.44
crystallization onset [°C]	†	†	†	†	†
crystallization energy [Jg^−1^]	†	†	†	†	†

† No crystallization observed in the range of the measurement.

**Table A5:** Calorimetric data of the 2gl_0.4PS_Tre [°C]. *n* = 2, values represent the mean value and the corresponding standard deviation.

	**RN**	**AN1.5**	**AN3.0**	**QN**	**CN**
T_g_ [°C]	104.46 ± 0.09	104.09 ± 0.04	104.08 ± 0.16	106.51 ± 0.23	100.11 ± 0.11
Δc_p_ [Jg^−1^K^−1^]	0.46 ± 0.07	0.47 ± 0.01	0.41 ± 0.01	0.41 ± 0.02	0.52 ± 0.01
$∆H_{\infty }\left(25\;{}^{\circ}C\right)$ [Jg^−1^]	36.59 ± 5.37	36.82 ± 0.97	32.06 ± 0.89	33.66 ± 1.70	39.36 ± 0.79
$∆H_{\infty }\left(40\;{}^{\circ}C\right)$ [Jg^−1^]	29.68 ± 4.36	29.83 ± 0.78	25.98 ± 0.72	27.47 ± 1.39	31.50 ± 0.64
crystallization onset [°C]	†	†	†	†	†
crystallization energy [Jg^−1^]	†	†	†	†	†

† No crystallization observed in the range of the measurement.

## Appendix B

The freezing steps conducted in the freeze-dryer were monitored by pressure and temperature sensors. Figure A1 displays the first 10 h of the RN and CN process, respectively. The freezing of the products is proven by the rise of temperature, which is caused from the heat of the ice nucleation process and emphasized in the figure by grey arrows. Absence of a second temperature peak is evidence that the samples were kept frozen after the ice nucleation without incomplete freezing or intermediate melting. Furthermore, the position of the peak demonstrates that the products of the RN process did not freeze during the equilibration step whereas the products of the CN process started to freeze immediately after the pressure drop and rise at approximately 1.5 h which introduces the ice fog in the chamber. The freezing step of the AN1.5 and AN3.0 process are equal to the RN process. The nucleation of the QN samples were not monitored due to practical reasons and safety during the freezing procedure. An absence of an ice nucleation in the freeze dryer indicates a successful freezing of the sample.

The actual cooling rate of the products themselves after freezing were determined by a linear fit during the ramp after nucleation resulting in a cooling rate of −0.85 K/min for RN, AN1.5 as well as AN3.0 and −0.75 K/min for CN samples.
